# Validation of the Diagnostic Score for Acute Lower Abdominal Pain in Women of Reproductive Age

**DOI:** 10.1155/2014/320926

**Published:** 2014-05-25

**Authors:** Kijja Jearwattanakanok, Sirikan Yamada, Watcharin Suntornlimsiri, Waratsuda Smuthtai, Jayanton Patumanond

**Affiliations:** ^1^Department of Surgery, Nakornping Hospital, Chiang Mai 50180, Thailand; ^2^Division of Gastrointestinal Surgery and Endnoscopy, Department of Surgery, Faculty of Medicine, Chiang Mai University, Chiang Mai 50200, Thailand; ^3^Department of Obstetrics & Gynecology, Nakornping Hospital, Chiang Mai 50180, Thailand; ^4^Department of Emergency Medicine, Nakornping Hospital, Chiang Mai 50180, Thailand; ^5^Clinical Epidemiology Unit, Clinical Research Center, Faculty of Medicine, Thammasat University, Pathum Thani 12120, Thailand

## Abstract

*Background*. The differential diagnoses of acute appendicitis obstetrics, and gynecological conditions (OB-GYNc) or nonspecific abdominal pain in young adult females with lower abdominal pain are clinically challenging. The present study aimed to validate the recently developed clinical score for the diagnosis of acute lower abdominal pain in female of reproductive age. *Method*. Medical records of reproductive age women (15–50 years) who were admitted for acute lower abdominal pain were collected. Validation data were obtained from patients admitted during a different period from the development data. *Result*. There were 302 patients in the validation cohort. For appendicitis, the score had a sensitivity of 91.9%, a specificity of 79.0%, and a positive likelihood ratio of 4.39. The sensitivity, specificity, and positive likelihood ratio in diagnosis of OB-GYNc were 73.0%, 91.6%, and 8.73, respectively. The areas under the receiver operating curves (ROC), the positive likelihood ratios, for appendicitis and OB-GYNc in the validation data were not significantly different from the development data, implying similar performances. *Conclusion*. The clinical score developed for the diagnosis of acute lower abdominal pain in female of reproductive age may be applied to guide differential diagnoses in these patients.

## 1. Background

Abdominal pain is one of the most common chief complaints of emergency department patients. It was the main symptom of 12.1% to 20.4% of noninjury visits to emergency departments of USA, and 16.8% to 17.8% of them were in severe conditions [1]. It is difficult to diagnose the causes of abdominal pain in some patients. Diagnosis of acute appendicitis, for example, was less accurate in young adult females than in males. The accuracies of diagnosis of acute appendicitis in young adult females were 71.7% to 75.3%, while the accuracies in male were 88.6% to 90.0% [2]. Diagnosis of acute lower abdominal pain in young adult females was particularly difficult due to overlapping symptoms of obstetrics and gynecological conditions with those of acute appendicitis. Negative appendectomies often occurred mostly from missed diagnoses of obstetrics and gynecological conditions [3].

CT scan improved accuracy in diagnosing appendicitis and can detect other causes of abdominal pain in female patients [4]. The use of CT scan can reduce negative appendectomies [5]. However, the universal use of CT scan for diagnosing appendicitis may not be cost-effective in global budget scheme reimbursement for healthcare [6].

Although ultrasound is not as accurate as CT scan, it also showed benefit in diagnosing acute lower abdominal pain [7, 8], especially for pregnant women and children, whom radiation is relatively contraindicated. However, ultrasound alone had low sensitivity in the diagnosis of appendicitis. Its sensitivity was not more than unaided-clinical judgment [9].

Clinical prediction rules, through which clinical findings were systematically applied to predict difficult clinical conditions [10], may be another approach for the diagnosis of acute lower abdominal pain in females of reproductive age. Alvarado's score, although intentionally developed for early diagnosis of acute appendicitis [11], has been studied for admission criteria [12] or criteria for CT scan [13]. However, appendicitis scores were not adequately applicable to abdominal pain in females of reproductive age, because they could not detect obstetrics and gynecological causes. We, therefore, developed a clinical scoring for the diagnosis of acute lower abdominal pain in these particular patients [14]. In this study, we aimed to validate our clinical scoring with patients in a different time period.

## 2. Method

### 2.1. The Scoring System

The score is comprised of simple clinical findings, laboratory results, and a constant. Item scores were assigned for guarding or rebound tenderness, pregnancy (either by clinical or urine pregnancy test), tenderness at right lower quadrant of abdomen, tenderness at left lower quadrant of abdomen, leukocytosis (white cell count ≥10,000/*μ*L), predominate neutrophil ≥75% in complete blood count, and a constant. The assigned scores and algorithm for diagnostic prediction were shown (Table 1). The item scores had both positive and negative values, which reflected an increase or a decrease in probabilities of the corresponding diagnoses when presenting with those clinical findings.

### 2.2. Validation Data

The setting hospital is Nakornping Hospital, a tertiary care hospital in Chiang Mai, Thailand. Validation data were extracted from the medical records of female patients aged 15–50 years who were admitted to surgical department or obstetrics and gynecology department during January and July 2009 with a chief complaint of acute lower abdominal pain within 14 days. Patients were classified into three groups upon their final professional diagnoses, which were (1) acute appendicitis (ICD10 code K-35); (2) obstetrics and gynecological conditions (OB-GYNc), including ectopic pregnancy (ICD10 code O-00), pelvic inflammatory disease (ICD10 code N70), and complicated ovarian cyst (ICD10 code N83); and (3) nonspecific abdominal pain (NSAP) (ICD10 code A09, K57, and R10 or other causes of abdominal pain). Study variables were age, marital status, duration of pain, presence of shifting of pain, nausea and vomiting, pregnancy, abnormal vaginal bleeding, presence of fever, systolic blood pressure, site of abdominal pain, presence of guarding or rebound tenderness from abdominal examination, result of complete blood count, and urine pregnancy test. Item scores were calculated and diagnostic prediction was performed for each patient. Final professional diagnoses in the medical records were considered as the reference standard for testing of the score accuracy.

### 2.3. Statistical Analysis

Patients' characteristics of the development data and the validation data were summarized. Score predicted diagnosis of each patient was compared with final professional diagnosis. Diagnostic indices were calculated in the validation data. The abilities to discriminate appendicitis and OB-GYNc, in terms of areas under the receiver operating curves of the two data sets, were compared with the test for equality of two ROC curves. The positive likelihood ratios for the diagnosis of appendicitis and OB-GYNc of the development data and the validation data were tested with chi-squared for homogeneity test. The probability curves of appendicitis score and OB-GYN score were estimated from logistic regression postestimation function on actual rates of appendicitis and OB-GYNc in the development data and validation data.

### 2.4. Ethics

The study was approved by the Ethical Committee of Nakornping Hospital and the Ethical Committee of the Faculty of Medicine, Chiang Mai University.

## 3. Results

The patients' characteristics of the derivation data and the validation data were similar (Table 2). Appendicitis was the most common diagnosis in both data sets (70.5% in development data and 65.2% in validation data). The final diagnoses of patients were shown (Table 3).

When comparing the score-predicted diagnoses and the final professional diagnoses in patients from the validation data, the score correctly diagnosed 24 of 33 NSAP patients (72.7%), 181 of 203 appendicitis patients (89.2%), and 46 of 66 OB-GYNc patients (69.7%). The overall accuracy of the score was 83.1% (251/302) (Table 4). The score had a sensitivity of 91.9%, a specificity of 79.0%, and a positive likelihood ratio of 4.39 for diagnosis of appendicitis. For the diagnosis of OB-GYNc, the score had a sensitivity of 73.0%, a specificity of 91.6%, and a positive likelihood ratio of 8.73, respectively. The diagnostic indices and their 95% confidence intervals were displayed (Table 5).

**Figure 1:**
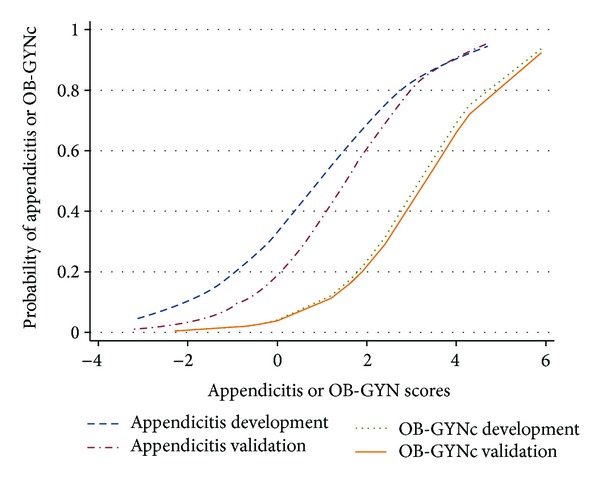
Estimated probabilities of appendicitis (dash and dash-dotted lines) and OB-GYNc (dot and solid lines) from actual rates of final diagnoses in development data set and validation data set.

When using the criteria in Table 1 for prediction of diagnoses, the performance of the score in discrimination of appendicitis in terms of ROC analysis and positive likelihood ratio in the validation data were not significantly different from those in the development data. The area under ROC curve for the discrimination of appendicitis and “nonappendicitis” was 0.855 in the validation data and 0.796 in the development data (*P* = 0.068). The positive likelihood ratios for diagnosis of appendicitis in the validation data and the development data were 4.39 and 2.97, respectively (*P* = 0.100). The areas under ROC curves for the discrimination of OB-GYNc and “non-OB-GYNc” were not different in the validation data and the development data (0.823 and 0.808; *P* = 0.706). The ROC areas of the development data reported in this study were different from those reported in our previous study because in previous study we reported the ROC areas of individual scores (appendicitis score for appendicitis and OB-GYN score for OB-GYNc), not as the whole algorithm like in this study. Similarly, the positive likelihood ratios for diagnosis of OB-GYNc were not significantly different in the validation data and the development data (8.73 and 12.94; *P* = 0.244) (Table 6). The estimate probability curves from actual rates in the development data and the validation data of appendicitis diagnosis from appendicitis score and OB-GYNc from OB-GYN score were shown (Figure 1).

## 4. Discussion

The present study was the second part of the previous study in clinical prediction rule for the diagnosis of acute lower abdominal pain in females of reproductive age [14]. In general, clinical prediction rule studies are comprised of derivation, validation, and impact studies, with an increase in the level of evidences in each phase [15]. Validation study is important before applying such clinical prediction rule into clinical practice because the results of prediction may not necessarily be reproducible in other settings or in the other time periods [16]. In this validation study, we found no significant differences in the prediction of diagnoses between the validation data and the development data. This could be explained simply by the fact that we conducted the study at the same setting as in the development of the diagnostic score; patients' characteristics and patterns of clinical practices were unlikely to be different from time to time.

Clinical scoring for the diagnosis of abdominal pain has been extensively studied for appendicitis [17–22]. There were relatively fewer studies for obstetrics and gynecological conditions [23–25]. However, those studied were applied for the diagnosis of only single disease (appendicitis, ectopic pregnancy, pelvic inflammatory disease, or adnexal torsion). The present diagnostic score has an advantage in inferring differential diagnosis of more than one condition, resembling routine clinical approach to patients. The main advantage of this score is triaging. It can guide emergency room physicians whether to admit the patients, and what specialties to consult. For example, a patient with appendicitis score and OB-GYN score equal to or less than zero, which diagnosis of NSAP is likely; this patient can probably be admitted to the observation room or discharged from emergency room and appointed to followup in the next 24 hours for a case with mild symptoms. The probability of appendicitis in this case would be approximately 20% or less; and the probability of OB-GYNc is very low (Figure 1). In addition, score-predicted probability in Figure 1 can also be applied for selective management. Patients with appendicitis score of 0–2 or OB-GYN score of 2–4, whose probabilities of appendicitis or OB-GYNc are approximately 20% to 60%, would be appropriate candidates for further investigations, such as ultrasound or CT, prior to admission. By triaging and selective management, the time spent in emergency department is expected to be less.

This study has several limitations. The obvious one is retrospective design of the study. Clinical signs and symptoms that were not documented either could be absent of such clinical findings or were not evaluated. The different observers may have different interpretations of physical examination, and clinical signs that change over time may not be well recorded.

Using of final professional diagnoses as the reference standard is another limitation. The problem of different follow-up times and different clinical judgments amongst doctors also leads to misclassification. These limitations can be reduced if a prospective validation study of the diagnostic scoring system is performed, with interobserver agreement of measurements, including standardized criteria for diagnostic indicators, objective criteria for final diagnosis of each condition, and standardized follow-up time.

The result of this study should be used with caution. Patients in our setting were mainly referred from smaller hospitals in Chiang Mai. Most of them needed to be admitted to either general surgery department or obstetrics and gynecology department. Different patients' characteristics and different patient flows in other settings would affect the accuracy of the scoring system. For example, myoma uteri complications such as necrosis or torsion were rare in our settings. In other hospitals where myoma uteri complications are major causes of acute lower abdominal pain, this diagnostic score may not be suitable for such settings. Applying this scoring system to different settings, different patterns of patients flow, could probably lead to misdiagnoses in some conditions. External validation in different settings should be performed prior to adoption into clinical practice in other settings. Further impact studies of the score to assess its impacts on multidimensions of clinical practice, such as time spent in emergency department, additional diagnostic value on top of unaided junior physicians' judgments, and time and cost of diagnosis, should be conducted in the future.

## 5. Conclusion

The clinical diagnostic score can triage appendicitis, OB-GYNc, and NSAP in female patients with acute lower abdominal pain. The diagnostic score can guide emergency department physicians for proper admissions and selective managements.

## Figures and Tables

**Table 1:** The scoring scheme for appendicitis and obstetrics-gynecological conditions (OB-GYNc) and the criteria used to guide diagnosis of abdominal pain caused by appendicitis, obstetrics, and gynecological conditions (OB-GYNc) or nonspecific abdominal pain (NSAP).

Predictors	Assigned score		Suggested diagnoses	Criteria
	Appendicitis score	OB-GYN score		
Guarding or rebound tenderness	1.9	0	Appendicitis	Appendicitis score > OB-GYN score and appendicitis score >0
Pregnancy	−1.7	2.4		
Leukocytosis (WBC ≥10,000/*μ*L)	1.5	0	OB-GYNc	OB-GYN score ≥ appendicitis score and OB-GYN score >0
Neutrophil ≥75%	1.3	1.6		
RLQ tenderness	1.5	0	NSAP	Appendicitis score ≤0 and OB-GYN score ≤0
LLQ tenderness	0	1.9		
Diarrhea	−1.4	−2.3		
Constant	−1.5	0		

RLQ: right lower quadrant; LLQ: left lower quadrant.

**Table 2:** Demographic and clinical characteristics of patients in the development data set and validation data set.

Characteristics	Development (*n* = 542)	Validation (*n* = 302)
Age (year)		
Mean (SD)	29.9 (10.7)	29.4 (10.3)
Single (%)	51.1	56.0
Duration of pain (hr)		
Mean (SD)	35.4 (41.4)	36.9 (47.2)
Shifting of pain (%)	29.3	16.6
Nausea and vomiting (%)	43.4	42.7
Abnormal vaginal bleeding (%)	5.7	4.6
Diarrhea (%)	8.5	8.9
Temperature ≥37.5°C (%)	28.3	35.3
Pulse rate (/min)		
Mean (SD)	89.6 (16.1)	89.7 (14.7)
Systolic blood pressure (mmHg)		
Mean (SD)	119.6 (16.6)	122.2 (16.6)
RLQ tender (%)	91.9	91.7
LLQ tender (%)	12.7	18.5
Guarding/rebound tenderness (%)	55.7	54.0
Hematocrit (%)		
Mean (SD)	37.0 (4.9)	36.5 (4.6)
WBC (/*μ*L)		
Mean (SD)	13266.1 (4928.0)	12811.3 (4639.4)
Neutrophil (%)		
≥75 (%)	56.9	62.3
Pregnant/positive pregnancy test (%)	10.5	12.6

**Table 3:** Final professional diagnosis of patients in the development data and validation data.

Diagnoses	Development (*n* = 542)	Validation (*n* = 302)
	n (%)	*n* (%)
Appendicitis	382 (70.5)	197 (65.2)
OB-GYNc	97 (17.9)	63 (20.9)
Ectopic pregnancy	48	34
Pelvic inflammatory disease	7	5
Complicated ovarian cyst	42	24
NSAP	63 (11.6)	42 (13.9)
Abdominal pain without specific diagnosis	31	20
Enteritis/colitis	21	15
Diverticulitis	5	4
Urinary tract infection	2	3
Radiation enteritis	2	
Twisted omentum	1	

**Table 4:** Diagnosis suggested by the scoring system and final professional diagnosis in the validation data.

Diagnosis suggested by scoring system	Final professional diagnosis			Total	Correct diagnosis (%)
	NSAP	Appendicitis	OB-GYNc		
NSAP	24	4	5	33	72.7
Appendicitis	10	181	12	203	89.2
OB-GYNc	8	12	46	66	69.7
Total	**42**	**197**	**63**	**302**	**83.1**

**Table 5:** Diagnostic indices (and 95% confidence interval) of the scoring system for appendicitis (versus nonappendicitis) and OB-GYNc (versus non-OB-GYNc) in the validation data (based on final professional diagnosis).

Diagnostic indices	Appendicitis (versus nonappendicitis)	OB-GYNc (versus non-OB-GYNc)
Sensitivity (%)	91.9 (87.1–95.3)	73.0 (60.3–83.4)
Specificity (%)	79.0 (70.0–86.4)	91.6 (87.4–94.8)
Receiver operating characteristic area	0.855 (0.811–0.898)	0.823 (0.765–0.881)
Positive likelihood ratio	4.39 (3.02–6.37)	8.73 (5.59–13.62)
Negative likelihood ratio	0.10 (0.06–0.17)	0.29 (0.20–0.44)
Positive predictive value (%)	89.2 (84.1–93.1)	69.7 (57.1–80.4)
Negative predictive value (%)	83.8 (75.1–90.5)	92.8 (88.7–95.7)

**Table 6:** Areas under receiver operating characteristic curves (AuROC) and positive likelihood ratios (and 95% confidence intervals) of the scoring system for appendicitis and OB-GYNc in the development and validation data.

Diagnosis	Development	Validation	*P*-value
Appendicitis			
AuROC	0.796 (0.751–0.841)	0.855 (0.811–0.898)	0.068
Positive likelihood ratio	2.97 (2.26–3.92)	4.39 (3.02–6.37)	0.100
OB-GYNc			
AuROC	0.808 (0.750–0.865)	0.823 (0.765–0.881)	0.706
Positive likelihood ratio	12.94 (7.91–21.17)	8.73 (5.59–13.62)	0.244
